# The Danish Turner Syndrome Cryopreservation study: PROTOCOL for a prospective cohort Study on reproductive outcomes after ovarian tissue cryopreservation in girls with Turner syndrome

**DOI:** 10.1186/s13023-025-04104-5

**Published:** 2025-11-17

**Authors:** Camilla Mains Balle, Signe M. Nørgaard, Christine M. B. Baggesgaard, Line B. Valsted, Mette H. Viuff, Rune W. Naeraa, Margit Dueholm, Mette-Cathrine R. Jakobsen, Iben Bache, Stine Gry Kristensen, Margit B. Fischer, Dorte Hansen, Katharina M. Main, Anette T. Pedersen, Casper P. Hagen, Claus H. Gravholt

**Affiliations:** 1 Department of Endocrinology and Internal Medicine, Aarhus University Hospital, Aarhus, Denmark; 2 Department of Clinical Medicine, Health, Aarhus University, Aarhus, Denmark; 3 Department of Pediatrics, Hans Christian Andersen Children’s Hospital, Odense University Hospital, Odense C, Denmark; 4 Department of Clinical Research, Faculty of Health Sciences, University of Southern Denmark, Odense, Denmark; 5 International Center for Research and Research Training in Endocrine Disruption of Male Reproduction and Child Health (EDMaRC), Copenhagen University Hospital – Rigshospitalet, Copenhagen, Denmark; 6 Department of Growth and Reproduction, Copenhagen University Hospital – Rigshospitalet, Copenhagen, Denmark; 7 Department of Molecular Medicine, Aarhus University Hospital, Aarhus, Denmark; 8 Department of Gynecology and Obstetrics, Aarhus University Hospital, Aarhus, Denmark; 9 Department of Paediatric and Adolescent Medicine, Aarhus University Hospital, Aarhus, Denmark; 10 Department of Clinical Genetics, Copenhagen University Hospital – Rigshospitalet, Copenhagen, Denmark; 11 Laboratory of Reproductive Biology, Department of Gynaecology, Fertility and Obstetrics, Copenhagen University Hospital – Rigshospitalet, Copenhagen, Denmark; 12 Department of Clinical Medicine, University of Copenhagen, Copenhagen, Denmark; 13 Department of Gynecology, Fertility and Obstetrics, Copenhagen University Hospital – Rigshospitalet, Copenhagen, Denmark

**Keywords:** Turner syndrome, Puberty, Fertility, Fertility preservation, Ovarian tissue cryopreservation, Premature ovarian insufficiency, primary ovarian insufficiency, Premature ovarian failure

## Abstract

**Background:**

Turner syndrome (TS) is a chromosomal disorder caused by complete or partial loss of one X chromosome, resulting in a high risk of premature ovarian insufficiency (POI) and infertility. While the underlying mechanisms are not fully understood, oocyte loss is known to begin in fetal life and continue throughout childhood, often leading to ovarian failure before the onset of puberty. Ovarian tissue cryopreservation (OTC) offers a potential method to preserve fertility in girls with TS, but the procedure remains experimental in this group. To date, only three cases of autotransplantation have been reported, and no live births have occurred in women with TS. Moreover, predictors of residual ovarian function and eligibility for OTC are poorly defined, and the long-term effects of unilateral oophorectomy in girls with already limited ovarian reserve are unknown.

**Objective:**

To investigate long-term reproductive outcomes following OTC in girls with TS.

**Methods:**

The Danish Turner Syndrome Cryopreservation Study is a nationwide, prospective cohort study with long-term follow-up, combining observational and interventional elements. Girls with TS aged 2–17 years are invited to participate. As ovarian reserve is depleted before adulthood in most girls with TS, all participants undergo ovarian function assessment to evaluate the likelihood of remaining viable follicles. If follicles are deemed present, unilateral OTC may be offered. The primary outcome is the number of pregnancies, miscarriages, and live births following autotransplantation of cryopreserved and thawed ovarian tissue in adulthood. Secondary objectives include identifying early predictors of POI, assessing the impact of oophorectomy on ovarian function and pubertal progression, and exploring molecular mechanisms of ovarian dysgenesis through genetic and epigenetic analyses of ovarian and peripheral tissues. Recruitment began in January 2024 and is ongoing at three university hospitals across Denmark. Follow-up is expected to continue until 2050.

**Conclusion:**

This study addresses a critical gap in fertility preservation for girls with TS by evaluating the feasibility and long-term outcomes of OTC. The findings will improve understanding of POI progression, refine selection criteria for OTC, and support evidence-based counselling for girls with TS and their families.

**Trial registration:**

ClinicalTrials.gov, NCT05740579. Registered 13 February 2023. https//clinicaltrials.gov/study/NCT05740579.

## Background

Turner syndrome (TS) is a chromosomal disorder affecting approximately 1 in 2000 live born females (ORPHA:881) [1]. It is caused by the complete or partial loss of one X chromosome, either in all cells or in a mosaic form with two or more different cell lines [2]. A hallmark feature of TS is premature ovarian insufficiency (POI), which is present in up to 95% of women with TS [3]. The exact mechanisms leading to ovarian failure in TS are not fully understood. However, an accelerated loss of germ cells begins in early fetal development [4–6], appears to continue throughout fetal life, and progresses rapidly during early childhood [7]. The underlying mechanisms involve increased apoptosis of oocytes and impaired formation of primordial follicles, resulting in compromised folliculogenesis during fetal life [5]. The accelerated loss of germ cells often leads to ovarian failure before the onset of puberty. However, a subset of patients retain sufficient ovarian function to undergo spontaneous pubertal development. More than one third of girls with TS develop signs of puberty, although only 15–35% experience spontaneous menarche [8–10]. Consequently, unassisted pregnancies are rare in women with TS, occurring in only 5–7% [11]. Notably, pubertal development, ovarian function, and fertility potential are highly dependent on karyotype. Girls with 45,X/46,XX mosaicism have the highest chance of retaining ovarian function, whereas girls with a 45,X karyotype are often born with fibrous streak gonads [12–14].

While TS is associated with multiple health challenges, infertility remains one of the most emotionally distressing, with the inability to have a genetically related child representing a major source of grief [15]. Ovarian tissue cryopreservation (OTC) presents a unique opportunity to preserve fertility in girls with TS before the complete depletion of germ cells. In contrast to oocyte vitrification, which requires post-pubertal maturation, OTC can be performed regardless of pubertal status, making it feasible even in infancy and early childhood. While OTC has proven successful in pediatric cancer patients treated with gonadotoxic therapy [16], its use in girls with TS remains experimental. Only a few TS cohorts have undergone OTC in Sweden [14, 17], the Netherlands [18], Denmark, the United Kingdom, and Australia [19]. Reports of subsequent autotransplantation are extremely limited, with only three documented cases in females with TS [17, 20]. Of these, only one reported a subsequent pregnancy, which unfortunately ended in an early miscarriage.

The Danish Turner syndrome Cryopreservation Study is a prospective cohort study designed to evaluate the long-term reproductive outcomes following OTC in childhood and subsequent autotransplantation in adulthood in women with TS. The study will also explore the natural history of TS, with particular focus on the progression of premature ovarian insufficiency (POI) and early predictors of POI during childhood. Furthermore, both genetic and epigenetic mechanisms underlying germ cell depletion will be investigated by integrating analyses of DNA methylation, gene expression, and proteomic profiles in ovarian and peripheral tissues.

## Methods

### Study design

**Fig. 1:**
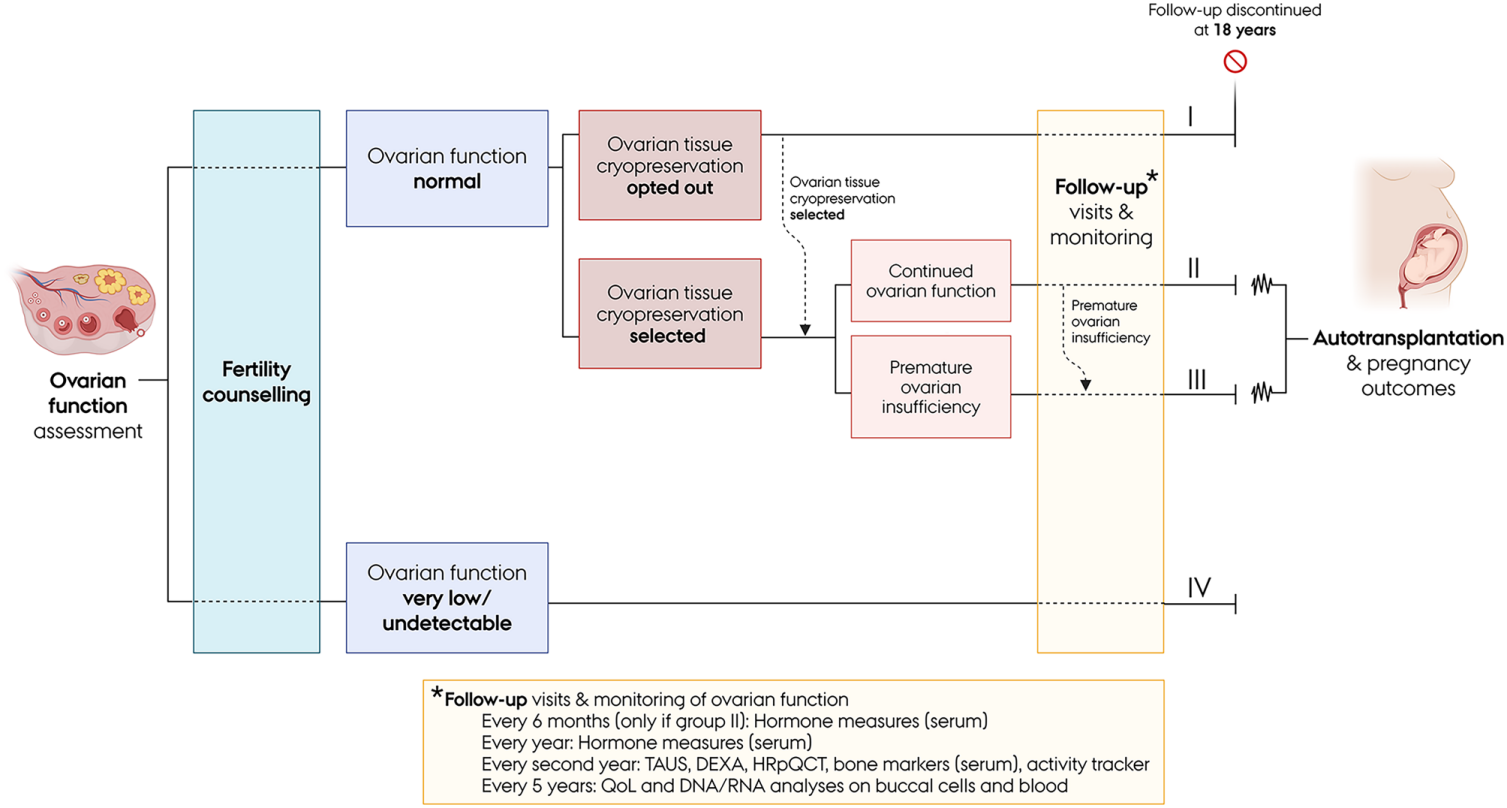
Overview of the Danish Turner cryopreservation study: design and follow-up protocol. Abbreviations: TAUS = transabdominal ultrasound; DXA = dual-energy X-ray absorptiometry; HRpQCT = high-resolution peripheral quantitative computed tomography; QoL = quality of life

This is a nation-wide, combined observational and interventional prospective cohort study with long-term follow-up. It is conducted across three university hospitals in Denmark and includes patients from all regions of the country. The study design is illustrated in Fig. 1. The study comprises four arms, defined by the initial assessment of the ovarian reserve and the remaining ovarian function following unilateral ovarian removal.

The trial protocol has been approved by the Scientific Ethics Committees for the Central Denmark Region (1-10-72-322-21). The study is registered at ClinicalTrials.gov (NCT05740579).

### Study period

Recruitment began in January 2024, and the study is expected to continue until 2050.

### Study population, inclusion and exclusion criteria

The study aims to include ovarian tissue for cryopreservation from a total of 100 girls with TS. Participants must be 2–17 years old with a confirmed diagnosis of TS, defined by a 45,X karyotype or a variant form such as 45,X/46,XX mosaicism, ring X mosaicism, or isochromosome X, in combination with typical clinical features of TS [21]. Exclusion criteria include severe cardiac disease contraindicating surgery and/or pregnancy, presence of Y chromosome material, and intellectual disability.

### Recruitment of participants and informed consent

Girls with TS and their parents will be informed about the study by their pediatric endocrinologist. Those who meet the inclusion criteria and express interest will receive detailed written information, tailored to the girl (if aged ≥ 15 years) and/or her parents. They will then be given as much time as needed to consider participation. Written informed consent must be obtained from all legal guardians. Girls aged ≥ 15 years must provide their own written consent in addition to that of their parents. Upon reaching the age of 18, participants will be asked to reconsent.

### Assessment of ovarian function

Since ovarian reserve is depleted before adulthood in most girls with TS, all participants undergo ovarian function assessment to evaluate the likelihood of remaining viable follicles. This approach aims to avoid unnecessary surgery and ensures that outcomes following OTC reflect the subgroup of girls with remaining viable follicles – and to whom OTC and later autotransplantation may be a feasible fertility preservation strategy.

Ovarian function is assessed through repeated measurements of serum hormone levels (including anti-Müllerian hormone (AMH), Inhibin B, gonadotropins (follicle stimulating hormone (FSH) and luteinizing hormone (LH)), and estradiol (E2)), clinical features of TS (such as pubertal development and current need for hormone replacement therapy), and imaging of the uterus and ovaries using transabdominal ultrasound (TAUS) and/or magnetic resonance imaging (MRI).

*Sufficient ovarian function for OTC was predefined as follows:*

- In prepubertal girls: age-adjusted AMH >-2 SD or FSH < + 2 SD, along with antral follicle count (AFC) >10th percentile on TAUS or MRI [13, 22, 23].
- In peri-/postpubertal girls: spontaneous pubertal development >-2 SD according to puberty nomograms [24], AMH, E2, and inhibin B >-2 SD, FSH and LH < + 2 SD, along with AFC >10th percentile on TAUS or MRI [13, 22, 23].

*Insufficient ovarian function for OTC was predefined as follows:*

- In prepubertal girls: AMH < -2 SD and FSH >+ 2 SD, along with streak ovaries or AFC < 10th percentile on TAUS or MRI [13, 22, 23].
- In peri-/postpubertal girls: absent or arrested pubertal development [24], primary or secondary amenorrhea, AMH, E2, and inhibin B < -2 SD, FSH >+ 2 SD, along with streak ovaries or AFC < 10th percentile on TAUS or MRI [13, 22, 23].

Following ovarian function assessment, the girls and their parents will discuss the results with both a gynecologist and a (pediatric or adult) endocrinologist with expertise in TS-related fertility. During this consultation, they will be informed about whether OTC can be offered, associated surgical risks, success rates of OTC in other patient groups, previous experiences with OTC in girls with TS, the risks associated with pregnancy, and alternative options for parenthood. They will also be informed about the increased risk of chromosomal abnormalities in potential children, and in the event of a pregnancy, the woman will be offered prenatal diagnostics (chorionic villus sampling). In cases where clinical, biochemical, and imaging findings do not provide a clear-cut assessment, cryopreservation may be considered following expert clinical assessment and a collaborative decision-making process with the family and the endocrinologist and/or gynecologist.

### Pre-operative assessment

According to the newly updated Clinical Practice Guidelines, recommendations against surgical intervention and/or pregnancy should be based on the patient’s individual cardiovascular risk profile [21]. Therefore, all participants deemed eligible for OTC will be examined by a trained cardiologist experienced in evaluating children and adolescents with TS. The examination includes transthoracic echocardiography (TTE), unless a recent examination is considered sufficient by the cardiologist. High-risk patients (and their parents) will be informed about the associated cardiovascular risks and will not be offered OTC if the risk of complications related to surgery or a future pregnancy is deemed too high.

### Additional baseline and follow-up assessments

In addition to blood samples and imaging performed for ovarian function assessment, a range of biological samples, imaging procedures, and clinical information is collected at baseline and at various follow-up intervals (Fig. 1). Buccal swabs are collected to screen for mosaicism using fluorescence in situ hybridization (FISH), as well as for DNA/RNA expression and methylation analyses. Additional blood samples are drawn for extended hormone profiling, assessment of bone turnover markers, and analyses of DNA/RNA expression and methylation. Bone health will be evaluated in all patients using dual-energy X-ray absorptiometry (DXA) to assess bone density and body composition. When practically feasible, high-resolution peripheral quantitative computed tomography (HRpQCT) will also be perfomed to examine bone microarchitecture. Free-living physical activity is tracked using a wearable activity monitor. Furthermore, quality of life is assessed using a questionnaire incorporating the WHO-5 Well-Being Index and the Satisfaction with Life Scale [25].

Examinations beyond standard care include DXA scans, HRpQCT (if offered), activity monitoring, buccal swab collection, MRI scan of the uterus and ovaries, and ovarian tissue cryopreservation (if offered). All procedures are provided free of charge, and participants (or their parents/legal guardians) will be reimbursed for travel expenses.

### Ovarian tissue cryopreservation (OTC)

The ovary is removed by laparoscopic unilateral oophorectomy, performed either at Copenhagen University Hospital – Rigshospitalet or at Aarhus University Hospital. The procedure is carried out by pediatric surgeons or gynecologists, depending on the patient’s age and maturity level. Ovaries removed at Aarhus University Hospital are placed in a specialized transport medium (Custodiol-HTK, Koehler-Chemie, Germany) and cooled during transfer (4–5 hours) to the Laboratory of Reproductive Biology at Copenhagen University Hospital – Rigshospitalet, where the tissue is processed and cryopreserved [26]. If the ovary is removed locally, it is transported in McCoy’s 5a medium with 25 mM HEPES at 37 °C to the same laboratory. Before cryopreservation, small biopsies (1–4 mm) are taken from both the ovarian cortex and medulla for analysis of follicle density, level of mosaicism, and assessment of DNA methylation status and RNA expression. If small antral follicles are present in the ovaries, follicular fluids with granulosa cells and immature oocytes will be aspirated and collected. In vitro maturation of collected immature oocytes will be attempted according to a previously developed protocol [27]. If surplus medullary tissue is available, enzymatic isolation and culture of preantral follicles will be attempted according to standard protocols [28].

### Ovarian tissue autotransplantation

Prior to pregnancy planning, fertility potential will be evaluated by a gynecologist or fertility specialist to assess the likelihood of spontaneous conception or indication for assisted reproductive technology (ART) and/or autotransplantation. If autotransplantation is indicated, preoperative screening is performed according to current guidelines. The procedure is carried out using minimally invasive surgery (laparoscopy or mini-laparotomy), where thawed ovarian tissue is transplanted into the remaining ovary or, if feasible, into peritoneal pockets. Follow-up includes hormonal assessment and transvaginal ultrasound to assess tissue activity. Further fertility planning will depend on signs of graft function and the patient will be treated according to a standard ART protocol.

## Outcome measures

### Primary outcome

- Number of pregnancies, miscarriages, and live births in women with TS following autotransplantation of cryopreserved and thawed ovarian cortical tissue, in relation to karyotype, age, prior hormonal status, and imaging findings.

### Secondary outcomes

- Primordial follicle density (number/mm³) in relation to karyotype, age, hormonal status, and imaging findings.
- Identification of markers and predictors of POI in girls with TS < 18 years following OTC.
- Epigenetic age acceleration before and after POI based on DNA methylation patterns in leukocytes.
- Association between DNA methylation, RNA expression and POI in leukocytes and ovarian tissue.
- Global gene expression and proteomic profiles in oocytes and granulosa cells from follicular fluid of small antral follicles.
- In vitro maturation potential and karyotype of oocytes from small antral follicles.
- TGF-beta signaling and steroidogenesis in theca cells, granulosa cells, and follicular fluid from small antral follicles.
- The effect of unilateral oophorectomy for OTC on residual ovarian function and pubertal development in girls with TS.

### Tertiary outcomes

- Uterine growth after OTC in girls with TS < 18 years not receiving HRT.
- The presence of somatic mosaicism, including differences in karyotype between buccal cells and peripheral lymphocytes, as well as the presence of germline mosaicism.
- Complications related to laparoscopic unilateral oophorectomy in girls with TS < 18 years.
- Bone mineral density, bone microarchitecture, and body composition before and after OTC.
- Quality of life in relation to ovarian function and OTC.
- Effect of endogenous estrogen on bone metabolism and cardiovascular risk markers after autotransplantation of cryopreserved and thawed ovarian cortical tissue.

## Statistical considerations and data analysis

All project data are entered into a centralized pseudo-anonymized REDCap database [29, 30], registered in the internal records of research projects maintained by the Central Denmark Region.

### Sample size calculation

Given the exploratory nature of this study, the live birth rate following OTC in women with TS is unknown. Power calculations are therefore based on data from female cancer survivors treated with gonadotoxic therapy. In this group, pregnancy rates following transplantation of thawed ovarian cortical tissue are approximately 50% and live birth rates around 35% [26, 31–33]. Spontaneous miscarriage occurs in an estimated 30–45% of TS pregnancies with autologous oocytes [11, 34]. Based on these figures, approximately seven patients would require OTC for one live birth to occur. Using the formula from Hulley et al. [35] for dichotomous outcomes (expected LBR of 0.25; a confidence interval width of 0.2; and a significance level (α) of 0.025) the required sample size is 72, corresponding to 18 expected live births. We aim to include 100 girls undergoing OTC. Data from participants lost to follow-up will be included where possible.

### Statistical analysis

Statistical analyses will be performed using STATA and SPSS. Methylation and RNA expression analyses will be conducted using R (EdgeR, DESeq2, Minfi).

Descriptive statistics will be used to analyze the number of ongoing pregnancies, miscarriages, and live births following autotransplantation of cryopreserved and thawed ovarian tissue, as well as the number of primordial follicles, time to pregnancy, and time to live birth. Continuous variables will be summarized as means with standard deviations (SD) or medians with interquartile ranges (IQR), depending on data distribution. Categorical variables will be summarized as percentages.

Associations will be assessed using Spearman’s correlation coefficient, including relationships between age at cryopreservation, hormone levels, and the number of primordial follicles.

## Discussion

Assessing ovarian activity and predicting future ovarian function in girls with TS remains challenging. In the context of OTC, a key difficulty is identifying which girls are most likely to benefit from the procedure. Although AMH levels are associated with karyotype, FSH/LH levels, and the presence of follicles [12, 14, 22, 36], and low AMH levels are associated with absent puberty and imminent POI in TS [13, 36], the overall predictive value of AMH remains uncertain. In one study of seven girls with TS undergoing ovarian stimulation and oocyte cryopreservation [37], no association was found between AMH levels, antral follicle count, and the number of oocytes retrieved. Remarkably, all participants had available oocytes for cryopreservation despite AMH levels as low as 3.05 pmol/L. In a Dutch cohort of girls with TS who underwent OTC [38], AMH was the best predictor of follicles present in the ovary removed for cryopreservation. Only 6% (4/67) of girls with undetectable circulating levels of AMH had ovarian follicles, and the density of follicles was very low [38]. Importantly, the chromosomal constitution of ovarian cells does not necessarily match that of peripheral lymphocytes or other tissues (e.g. buccal cells). Thus, the proportion of affected cells in peripheral blood is not always predictive of the remaining pool of primordial follicles. This may explain the occasional spontaneous pregnancies in women with complete 45,X monosomy [39]. Peek et al. [40] analyzed 46 oocytes from 10 women with TS and found that while 90% of the oocytes were correctly tetraploid (arrested in prophase of meiosis I), the surrounding granulosa cells were predominantly 45,X. These findings imply that although AMH levels and karyotype are useful indicators of ovarian function and the presence of follicles, they are not sufficient as stand-alone predictors. Follicles may still be present, and potentially usable for OTC, even in individuals with low AMH levels or complete 45,X monosomy in peripheral lymphocytes. This underscores the need for further research into ovarian mosaicism and the identification of more reliable biomarkers for assessing ovarian function and determining eligibility for OTC in girls with TS.

The timing and mechanisms underlying the loss of ovarian function in TS are not fully understood. Of particular interest are X-linked genes such as *KDM6A*, *USP9X*, and *ZFX*, which play critical roles in germ cell development and escape X inactivation. These genes have been found to be either differentially methylated [41] or differentially expressed [42] in adult women with TS compared to 46,XX controls, based on analyses of DNA from leukocytes or fibroblasts. However, as gene expression and methylation patterns are highly tissue-specific, it is essential to replicate these analyses in ovarian tissue. We plan to perform genomic analyses on a portion of the excised ovarian tissue to gain further insight into the molecular mechanisms underlying ovarian dysgenesis in TS.

Since most individuals with TS who have undergone OTC have not yet reached an age at which pregnancy is typically pursued, long-term reproductive outcomes remain unknown. To date, no live births have been reported in women with TS following autotransplantation of cryopreserved and thawed ovarian cortical tissue. One case report has described a clinical pregnancy in a woman with TS following re-implantation of autologous ovarian cortex cryopreserved shortly after spontaneous puberty; unfortunately, it resulted in an early miscarriage at approximately seven weeks of gestation [20]. As such, the procedure remains experimental in this population, and the absence of clinical evidence poses important ethical challenges and complicates the counselling of eligible girls and their families. In prepubertal girls, OTC requires laparoscopic unilateral oophorectomy, which carries a small risk of surgical complications, including bleeding and infection. Moreover, if autotransplantation does not lead to pregnancy, the procedure may generate false hope and increase the risk of future distress [43, 44]. A further concern is that removal of one ovary at a young age may accelerate the depletion of an already severely compromised ovarian reserve. Although studies in the general population suggest that unilateral oophorectomy only advances the age of menopause by approximately 1–2 years [45, 46], these findings may not be generalizable to girls with TS, who typically undergo oophorectomy at a much earlier age and are inherently at risk of POI. In a recent prospective follow-up study of 28 girls with TS who underwent OTC, van der Coelen et al. [47] investigated whether the natural progression of puberty was affected. The authors argued that pubertal development proceeded largely as expected based on age, karyotype, and pre-OTC hormonal status. However, the longest follow-up was only 6.6 years, and several participants had not yet reached the age at which the onset – or absence – of puberty could be evaluated. Interestingly, in most girls, AMH levels declined during the first year following OTC, before stabilizing or increasing – in some cases exceeding pre-OTC levels. The pattern was most pronounced in prepubertal girls. Further research is needed to elucidate the longitudinal trends of AMH following OTC and the clinical implications. Taken together, these factors underscore the need for caution and comprehensive counselling when offering experimental fertility preservation in TS, particularly considering the current lack of evidence on long-term outcomes following OTC.

The Danish Turner Syndrome Cryopreservation Study represents an important step toward understanding the potential and limitations of OTC in TS. By prospectively following girls with TS across the full spectrum of ovarian function – including those not deemed eligible for OTC – the study will provide valuable insights into the progression of ovarian insufficiency, as well as long-term reproductive outcomes following OTC and subsequent autotransplantation. These findings will help refine patient selection, enhance clinical counselling, and support evidence-based decision-making regarding fertility preservation in this population.

## Data Availability

Not applicable.
